# Therapy of Dysphagia by Prolonged Pharyngeal Electrical Stimulation (Phagenyx) in a Patient with Brainstem Infarction

**DOI:** 10.3390/brainsci10050256

**Published:** 2020-04-28

**Authors:** Cristina Florea, Christine Bräumann, Christine Mussger, Stefan Leis, Larissa Hauer, Johann Sellner, Stefan M. Golaszewski

**Affiliations:** 1 Department of Neurology, Christian Doppler Medical Center, Paracelsus Medical University, 5020 Salzburg, Austria; 2 Department of Psychiatry, Psychotherapy and Psychosomatic Medicine, Christian Doppler Medical Center, Paracelsus Medical University, 5020 Salzburg, Austria; 3 Department of Neurology, Landesklinikum Mistelbach-Gänserndorf, 2130 Mistelbach, Austria; 4 Department of Neurology, Klinikum rechts der Isar, Technische Universität München, 81675 München, Germany; 5 Karl Landsteiner Institute for Neurorehabilitation and Space Neurology, 5020 Salzburg, Austria

**Keywords:** dysphagia, ischemic stroke, pharyngeal electrical stimulation, treatment, rehabilitation

## Abstract

Dysphagia after stroke impacts quality of life and is a risk factor for respiratory infections. Patients frequently require prophylactic measures including nasogastric tube or percutaneous endoscopic gastrostomy. Until recently, therapy for dysphagia was limited to training with a speech and language specialist. Intraluminal pharyngeal electrical stimulation (PES) is a new technique that stimulates the pharyngeal sensory afferents to the higher swallowing center in cortex. The clinical trials published to date involved stimulation for 10 minutes over three days. We present a case of brainstem infarction with severe dysphagia in a 53-year-old woman with preserved cognitive functions. For airway protection, she had a surgical tracheotomy. The initial swallowing training achieved slight improvements, but stagnated after three months so PES was tried. Under good PES tube tolerance, a prolonged and repeated stimulation protocol was administered, with the main purpose of relieving her of the tracheal tube. Although the swallowing improved, she stayed tube-dependent with minimal attempts with puréed food during therapy, and could not be decannulated. Further studies are required to assess the value of this promising approach for the treatment of dysphagia.

## 1. Introduction

Swallowing is a process dependent on a central pattern generator, located in the medulla oblongata, in an area corresponding to the solitary tract nucleus, and on the cortical swallowing area, situated in the frontal lobe on both sides with a side-dominance unrelated to handedness [1]. Swallowing can be initiated voluntarily and involuntarily, triggered by central input (cortical) or peripheral sensory afferents (through cranial nerves V, VII, X–XII), respectively [2]. Pharyngeal electrical stimulation (PES) is a novel technique intended to activate the peripheral sensory afferents to the central pattern generator and to the cortical swallowing area by a nasogastric tube with electrodes around it (in the tube portion that lies in the pharyngeal area). The intensity of the stimulation is dependent on the patient’s sensory and maximum tolerated thresholds. The current is administered by a speech and language specialist using a base station to control the intensity and duration of the stimulation [2]. Previous studies focused on the application of PES in supratentorial stroke, which from a neurophysiological point of view may hold a greater potential for treatment response because there is healthy brain tissue in the contralateral hemisphere to take over when stimulated. If the brainstem-swallowing center where all the fibers converge, however, is affected, there is probably less potential for stimulus-induced neuroplasticity.

In 2010 Jayasekeran et al. published a dose–response study of 22 patients, which established a stimulation protocol on three consecutive days with 10 min of stimulation per day [3]. This protocol was used in some of the later studies.

Even with this short stimulation protocol, most of the studies showed improvements in swallowing, tendencies towards shorter hospital stay and much higher rates of decannulation in tracheotomized patients without serious adverse effects and without significant difference between stimulation and control groups regarding respiratory infections, impairment or death [4,5,6,7]

We applied PES in a patient with infratentorial infarction and used a prolonged and repeated stimulation protocol. The stimulation was applied in the chronic phase of the infarction, and swallowing was evaluated with flexible endoscopic evaluation of swallowing (FEES).

## 2. Case Report

**Figure 1:**
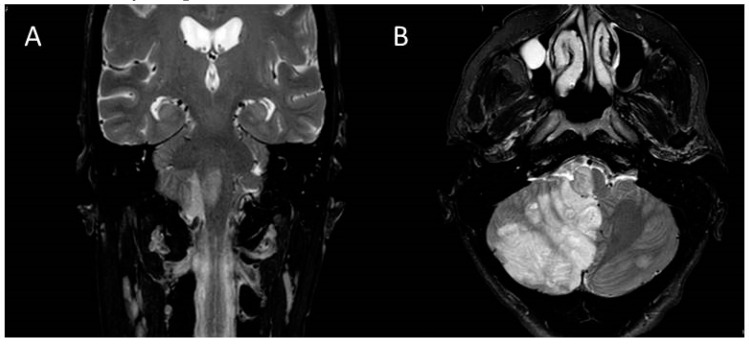
Coronal (**A**) and (**B**) transversal T2-weighted magnetic resonance imaging (MRI) which depicts ischemic stroke in the right cerebellar hemisphere, the upper medulla oblongata and the pontomedullary junction.

We report on a 53-year-old woman who was admitted to the early neurorehabilitation unit 36 days after she had suffered an ischemic stroke of the brainstem and cerebellum (Figure 1). The etiology of the stroke was thrombosis of both vertebral arteries (right complete, left incomplete), occlusion of the lower basilar artery over a length of 2.5 cm and occlusion of the right posterior inferior cerebellar artery (PICA). Her initial symptoms had been vertigo, diplopia, disturbed swallowing, hemiataxia of the right extremities and hemiparesis of the left side. 

Because of an acute respiratory insufficiency due to aspiration pneumonia, she had been in the intensive care unit (ICU) in the first weeks after the infarction with intermittent mechanical respiration. Afterwards, she was tracheotomized (first dilatative tracheotomy, after two weeks then revised on surgical tracheotomy) for airway protection and received a percutaneous endoscopic gastrostomy (PEG) for enteral feeding. After the acute respiratory insufficiency, the left hemiparesis worsened to hemiplegia, but no new infarctions were seen on magnetic resonance imaging (MRI). While in the ICU, a first PES was tried but not tolerated (just a single stimulation for 10 min, then the tube was self-removed by the patient).

She received standard physiotherapy, occupational therapy and logopedic training. Hypersalivation and bronchotracheal secretion were managed medically with scopolamine, amitriptyline, and chemodenervation with botulinum toxin in the parotid gland. One hundred days after her stroke, the improvements in dysarthria and dysphagia slowed down, so another attempt with PES was considered of possible benefit.

For the evaluation of the swallowing status, we used FEES and the clinical logopedic evaluation with modified Evan’s blue dye test [8]. The parameters assessed by these tests included penetration aspiration scale (PAS) [9], functional oral intake scale (FOIS) [10] and Bogenhausen dysphagia score (BODS) [11], as well as the swallowing frequency in the resting state. The terms penetration and aspiration are used to describe when the saliva or bolus penetrate in the larynx up to the vocal cords, or are aspirated below the vocal cords, respectively.

A FEES examination before the PES treatment showed aspiration and deep penetration of saliva without successful removal of the secretion through coughing. The examination with ½ teaspoon (tsp.) of purée showed a deep penetration with contact to the vocal folds and removal from the airway (penetration aspiration scale PAS 4). As the patient was tube-dependent with no oral intake, she had a FOIS (functional oral intake scale) score of 1. She also had a paresis of the right vocal cord. She had several dysphagia patterns during the first logopedic testing. These included a hypoglossus paresis, a velum paresis on the right, a facial palsy on the right, disturbed opening of the upper esophageal sphincter and consecutive postdeglutitive residues, wet voice, a missing swallowing reflex trigger, reduced cleaning mechanism, oral saliva retention, hypernasality and intra-oral and extra-oral dysfunction of sensibility.

The commercial device we used was Phagenyx^©^ (Phagenesis Ltd, Manchester, UK). After receiving informed consent from the patient, the nasogastric tube was introduced by a doctor according to the specifications of the device (very similar to a normal nasogastric tube). The speech and language specialist then applied the stimulation in the patient’s training sessions. After stimulation, whenever possible, logopedic training followed (less than 50% of the time, due to restricted availability of speech and language specialists). 

**Figure 2:**
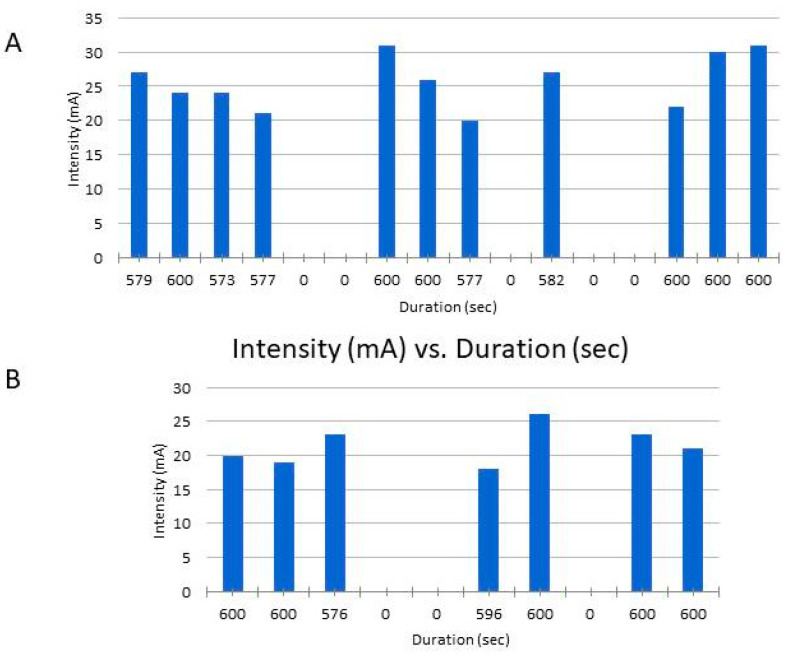
Stimulation intensity and duration during the first (**A**) and second (**B**) stimulation protocol.

**Figure 3:**
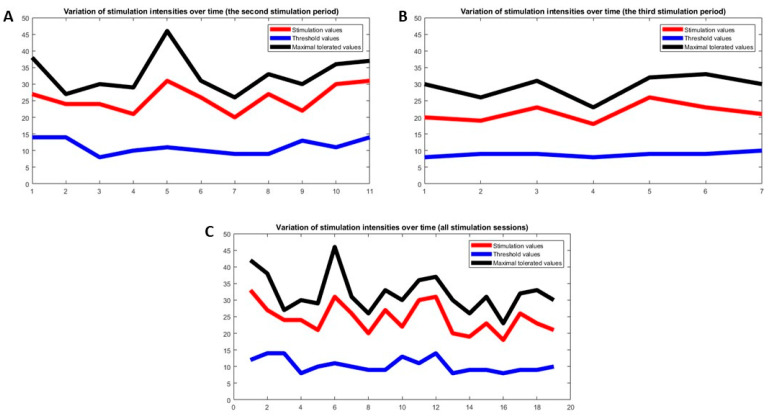
Stimulation intensities over time. (**A**) Second stimulation period with high stimulation intensities two months after the first stimulation period in the acute phase. (**B**) Third stimulation period with lower stimulation intensities three months after the first stimulation period. (**C**) Stimulation intensities of all three stimulation periods over time with decreasing stimulation intensities.

The patient tolerated the therapy very well and there appeared to be an improvement in the phonation and swallowing activity, so that PES was prolonged for a total of 11 days over a period of 16 days (no stimulation on weekends and holidays) (Figure 2 and Figure 3). This decision was taken with the consensus of doctors, speech and language specialists, the patient, a technical device expert and nurses. The patient was free at every point to interrupt the PES and ask for the tube to be removed.

The clinical–logopedic evaluation after this PES session showed improvements in phonation and sensibility of the pharynx with increased frequency of coughing. The modified Evan’s blue dye test showed persistent aspiration. The FEES, performed one week after the last day of stimulation, showed less saliva retention, as well as minor improvements in sensibility of the pharynx and in phonation. The examination with ½ tsp. of purée showed a penetration with successful laryngeal clearing (PAS 2). Because of persistent saliva aspiration, the patient could not yet be decannulated [8]. As she stayed tube-dependent with no oral intake, she had a FOIS (functional oral intake scale) score of 1.

After an interval of three weeks (while standard therapies continued), a new PES was administered, this time for seven consecutive days over an interval of 10 days (Figure 2B). After this second stimulation session, the aspiration improved clinically, but not enough to remove the tracheal tube. In this regard, her swallowing frequency in the resting state increased (three times in 10 min) and she was mainly able to control her saliva without endotracheal suctioning. The modified Evan’s blue dye test showed no aspiration of saliva. These clinical improvements allowed the continuous (even during the night) de-blocking of the tracheal cannula (no cuff to prevent saliva from running down the trachea) and no more suctioning of the tracheal tube was necessary. Finally, the cannula was also plugged continuously, so the patient breathed nasal–oral and not through the tracheostoma.

In the FEES control, two weeks after the last stimulation, a further improvement was noted: saliva could be swallowed without aspiration, but ½ tsp. of purée was aspirated. However, the patient had a strong cough reflex and with the chin-tuck maneuver she swallowed again without aspirating (PAS 1). This was a step forward and it allowed the gustatory probation of puréed foods during logopedic therapy (FOIS 2). As the tracheal cannula had been plugged during the day and night for more than a week (no breathing through the cannula, only nasal–oral), it could be changed to an unblocked one. Tracheal suctioning was not required anymore and she was able to manage her oral secretions with swallowing maneuvers such as chin-tuck and frequent coughing (Table 1). The patient fulfilled the decannulation criteria according to Muhle et al. but we observed several panic episodes due to anxiety for asphyxia [12]. Therefore, decannulation was postponed to the end of the whole rehabilitation process four weeks after the end of the third stimulation period.

## 3. Discussion

In 1997, Dr. Hamdy and his team first proved that stimulating the afferent pathways of swallowing (cranial nerves V and X) facilitates the swallowing cortex [13]. In a larger proof-of-concept study by Fraser et al. [14], the parameters for stimulation were established (5 Hz frequency, 10 minutes’ duration, intensity as high as possible—depending on the maximum tolerated by the patient). They also mapped the pharyngeal cortex by motor-evoked potentials and showed that PES raised the excitability and the size of the swallowing cortex. In addition, a response to PES was visible on fMRI. In the same study, PES was administered to a small group of stroke patients with dysphagia (10 PES, 6 control) and the increase in cortical excitability was associated with clinical improvements in swallowing. 

Further studies concentrated on proving the clinical benefits of PES and on refining and standardizing the stimulation protocol [3,15]. Jayasekeran et al. induced by repetitive transcranial magnetic stimulation (rTMS) a virtual lesion in the cortex of healthy volunteers and reversed it with PES [3]. The same article also includes the dose–response study that established the ‘standard’ protocol of 10 minutes a day, for three consecutive days, as well as a small placebo-controlled trial in 28 patients with acute stroke. In this trial, PES reduced the aspiration, improved the feeding status and was associated with shorter hospital stay. The dose–response study was completed by 22 stroke patients with dysphagia. Six patients were not stimulated; the other 16 were divided into four treatment groups: 3, 5, 9 and 15 stimulations (once a day for three or five days, and three times per day for three or five days). The best results (reduction in aspiration) were noticed in the once-a-day and three-day-long stimulation groups, but the difference between these results and the three times per day (3×/d) or five-day-long (5-d-long) stimulation groups were not statistically significant. Not significant also were the differences between 3×/d or 5-d-long groups versus the control group. This situated the 3×/d and 5 d groups in the middle of the best results scale (between control and 1×/d, 3 d), but it also shows how very small the differences between groups were. However, these unreliable results were accepted and transferred to other studies, where only the 1×/d, 3-d-long stimulation was applied [5,7,16]. To date, very little information about longer or repeated PES protocols has been published [6]. In this regard, Muhle et al. used PES in 23 tracheotomized stroke patients who could not be decannulated due to severe and persisting dysphagia [12]. In that study, 61% of participants could be decannulated after the first treatment cycle. Since our patient tolerated the PES very well and was highly motivated to improve her swallowing so that she could be decannulated, we prolonged the stimulation protocol and then repeated it.

Another noticeable weakness of the studies to date is that most of the included patients had hemispheric, supratentorial stroke. Our patient had a stroke of the brainstem and cerebellum, which implies that her dysphagia was due to a lesion of the central pattern generator and/or corticonuclear tracts to the cranial nerves (or a lesion of the nuclei themselves) that innervate the oropharyngeal musculature. Her progress suggests that PES may also improve swallowing in this type of lesion. The aforementioned studies focused on applying the stimulation as soon as possible after stroke (mostly in the subacute (1 week–1 month) phase). Our case report shows that improvements in swallowing under PES can also be achieved in later, chronic stages of the infarction. 

## 4. Conclusions

In conclusion, the pharyngeal electrical stimulation is a promising therapeutic approach for stroke patients with dysphagia, be it a supra- or infratentorial stroke, improving the prospects of decannulation for tracheotomized patients, as well as the chances of transition to oral food intake. However, more studies with different (longer, repeated or later applied) stimulation protocols and including infratentorial strokes are necessary to determine the optimal therapy for each category of patients.

## Figures and Tables

**Table 1:** Chronological presentation of the results from evaluation tests and scores before and after pharyngeal electrical stimulation (PES).

Test	Day 72 after Stroke	PES 1 (Days 100–115 after Stroke)	Day 123 after Stroke	PES 2 (Days 135–138 after Stroke)	Day 151 after Stroke
FEES	- massive aspiration of saliva - cannula should be blocked (cuffed) in the night - no oral food/drink intake		- retention of saliva in the valleculae, piriform recesses and retrocricoidally, with tendency towards aspiration - try de-blocked cannula in the night - no oral food/drink intake - try gustatory stimuli in logopedic therapy		- saliva swallowed without aspiration - ½ tsp. of purée aspirated with strong cough reflex, no aspiration with chin-tuck position - switch to uncuffed cannula - no oral food/drink intake - continue gustatory purée with chin-tuck in logopedic therapy
PAS	4 (½ tsp.)		2 (½ tsp.)		1 (½ tsp.)
FOIS	1		1		2
BODS totalBODS 1/BODS 2	BODS 14 (6 + 8)		BODS 12 (5 + 7)		BODS 11 (4 + 7)
Swallowing frequency in resting state	0×/10 min		1×/10 min		3×/10 min
	Before 1st PES		After 1st PES		After 2nd PES
Modified Evan’s blue dye test	Persistent aspiration		Persistent aspiration		No aspiration

PAS: penetration aspiration scale [9]; FOIS: functional oral intake scale [10]; BODS: Bogenhausen dysphagia score [11].
